# Amiodarone Treatment in the Early Phase of Acute Myocardial Infarction Protects Against Ventricular Fibrillation in a Porcine Model

**DOI:** 10.1007/s12265-018-9861-6

**Published:** 2019-01-07

**Authors:** Stefan M. Sattler, Anniek F. Lubberding, Lasse Skibsbye, Reza Jabbari, Reza Wakili, Thomas Jespersen, Jacob Tfelt-Hansen

**Affiliations:** 1 Department of Cardiology, Heart Centre, Copenhagen University Hospital, Rigshospitalet, Blegdamsvej 9, 2100 Copenhagen, Denmark; 2 Department of Medicine I, University Hospital Munich, Campus Grosshadern, Ludwig-Maximilians University Munich (LMU), Munich, Germany; 3 Department of Biomedical Sciences, Faculty of Health and Medical Sciences, University of Copenhagen, Copenhagen, Denmark; 4 Department of Exploratory Toxicology, H. Lundbeck A/S, Valby, Denmark; 5 Department of Cardiology and Vascular Medicine, West-German Heart and Vascular Center Essen, University of Essen Medical School, University Duisburg-Essen, Essen, Germany; DZHK (German Centre for Cardiovascular Research), Partner Site Munich, Munich Heart Alliance (MHA), Munich, Germany; 6 Department of Forensic Medicine, Faculty of Medical Sciences, University of Copenhagen, Copenhagen, Denmark

**Keywords:** Ventricular fibrillation, Amiodarone, Acute myocardial infarction, Sudden cardiac death, Animal model

## Abstract

Ventricular fibrillation (VF) occurring in the first minutes to hours of acute myocardial infarction (AMI) is a frequent cause of death and treatment options are limited. The aim was to test whether early infusion of amiodarone 10 min after onset of AMI reduced the incidence of VF in a porcine model. Eighteen female Danish landrace pigs were randomized to a control and an amiodarone group. AMI was induced by ligation of the mid-left anterior descending artery for 120 min followed by 60 min of reperfusion. VF occurred in 0/8 pigs treated with amiodarone compared to 7/10 controls (*P* < 0.01). Amiodarone treatment prolonged RR intervals, reduced dispersion of action potential duration in the infarcted area and mean number of ectopic beats. No negative effects on cardiac output and blood pressure were observed with amiodarone. Amiodarone qualifies as a potential drug candidate to prevent VF in the first minutes to hours of AMI.

## Introduction

Coronary artery disease and its ultimate consequence, acute myocardial infarction (AMI), is responsible for approximately 75% of all sudden cardiac death (SCD) in the Western world. Moreover, half of all deaths due to coronary artery disease occur suddenly with ventricular fibrillation (VF) or ventricular tachycardia (VT) degenerating into VF, as the most common underlying arrhythmia [1].

Cardiac arrest caused by VF in the early stages of AMI often occurs either within minutes after the onset of clinical symptoms or as the first symptom of AMI [2]. Even if medical contact is established before VF occurs, the 30-day survival rate in patients experiencing VF prior to or during primary percutaneous coronary intervention (PPCI) is 79% compared to 94% in patients without VF [3]. The GEVAMI study, our own Danish nationwide cohort study, determined the incidence of VF in 660 consecutive ST-elevation myocardial infarction (STEMI) patients to be 11.6% prior to PPCI [4]. Results from the recent French e-MUST study determined the incidence of VF after the STEMI diagnosis was given by the EMS to be 4.2% [5]. The APEX-AMI trial showed that mortality rate was twofold higher in patients with VT/VF before PPCI was completed and fivefold higher in patients with VT/VF occurring after PPCI compared to AMI patients without VT/VF [6], emphasizing the severity of this condition and the importance of prophylactic pharmacological treatments.

However, treatment options are hitherto limited and current guidelines do not recommend prophylactic antiarrhythmic drug treatment, as their use during AMI is often associated with further arrhythmias and hemodynamic depression [7]. An overview of relevant studies is given by Dagres & Hindricks [8]. In brief, a meta-analysis including 37 randomized controlled trials involving 11,948 patients on the prophylactic use of the sodium channel blocker lidocaine (class Ib antiarrhythmic agent) in patients with known or suspected AMI showed very little or no effect on mortality or VF. Further, the use of the class Ic antiarrhythmic drugs encainide and flecainide was associated with increased arrhythmia and SCD. Beta blockers are known to relieve ischemia and likely to reduce in-hospital VT/VF but their effectiveness on the prevention of VT/VF in the early phase of AMI has not been evaluated in randomized trials.

Amiodarone is a commonly used antiarrhythmic class III agent with a complex pharmacological profile. Originally developed as an antianginal drug five decades ago, it is the most effective anti-arrhythmic compound for a variety of different arrhythmias, and has shown to be relatively safe and improve overall survival rates in patients with ischemic heart disease [9]. Acute amiodarone treatment inhibits multiple ion-channels, predominantly inward Na^+^ and Ca^2+^ currents and outward K^+^ currents, and acts as a non-competitive beta-blocking agent [10].

Previous animal studies investigating amiodarone during AMI showed effects on a variety of electrophysiological parameters, like a prolonged effective refractory period (ERP) and an increased VF threshold without effecting defibrillation threshold in pigs [11]. In dogs with AMI and early onset VF, amiodarone treatment increased the defibrillation success rate compared to epinephrine and lidocaine alone from 13 to 88% [12]. Furthermore, it reduced the frequency of premature ventricular contractions (PVC) and the VT rate [13].

In humans, no evidence of the use of amiodarone in the acute setting of AMI, within the first 60 min after onset and before PPCI, is available since randomized trials mainly looked at unselected out-of-hospital cardiac arrest (OHCA) cohorts. A recent controlled randomized trial on 3026 patients studied the use of amiodarone or lidocaine and failed to show an improved survival in OHCA patients compared with placebo [14]. No randomized trials in humans, investigating treatment with amiodarone in the acute setting of AMI to prevent VT/VF in the early phase before or during PPCI, exist.

The aim of this study was to assess whether the use of a clinically relevant dose (3 mg/kg) amiodarone infused 10 min after onset of AMI could reduce the incidence of VF within 2 h of coronary occlusion and 1 h of reperfusion in a porcine model. The time interval of 10 min was chosen to account for the time emergency medical services need to arrive. Since amiodarone has positive effects on VF threshold and reduces the amount of ventricular arrhythmias, our hypothesis was that amiodarone can prevent VF in these animals without affecting the hemodynamic stability.

## Material and Methods

### Animals

Eighteen female Danish landrace pigs weighing 47–54 kg were randomly assigned to receive either amiodarone (*n* = 8) or vehicle (*n* = 10). A higher number of animals were randomized to the control group due to expected higher VF rates. Randomization took place the day before the experiment was conducted. All experiments were performed under the animal license number (2015-15-0201-00613) authorized by the Danish Animal Inspectorate in accordance with EU legislations for animal protection and care.

### Surgical Preparation

Pigs were pre-medicated, intubated, and anesthetized with continuous intravenous fentanyl 0.1 µg/kg/h (Fentanyl-Hameln 50 μg/ml, Hameln, Germany) and propofol 12.5 mg/kg/h (Propolipid 10 mg/ml, Fresenius Kabi AB, Uppsala, Sweden). Heparin (10,000 IE; Heparin LEO 5000 IE/ml, Leopharma A/S, Ballerup, Denmark) was administered and a Swan Ganz catheter (Swan-Ganz VIP, Edwards Lifesciences, Irvine, USA) to measure cardiac output (CO) was placed into the pulmonary artery.

**Fig. 1:**
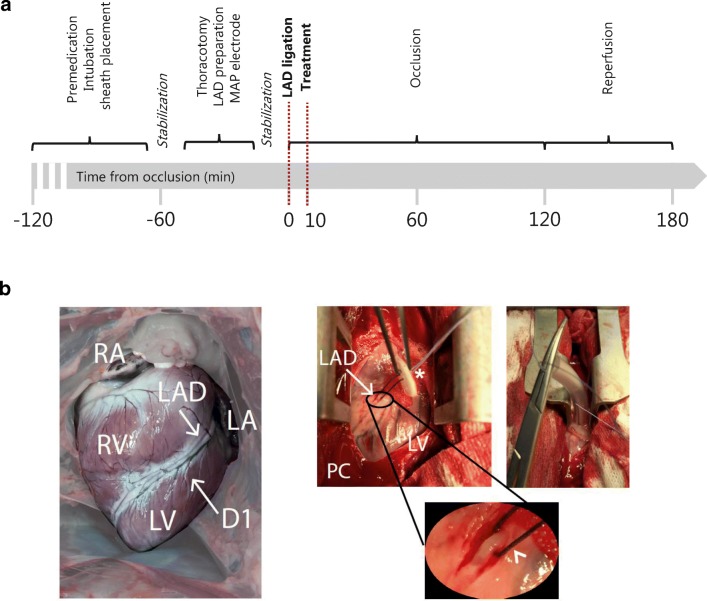
Experimental setup. **A** After premedication and implantation of the venous and arterial sheaths, the animal was left for stabilization. A mid-sternal thoracotomy was performed, the mid-left anterior descending artery (LAD) was dissected free and a silk snare was placed. Monophasic action potential (MAP) electrodes were placed and after a stabilization phase the snare was tightened around the LAD. Ten minutes after ligation, 3 mg/kg body weight amiodarone was given intravenously. After 120 min of occlusion, the snare was opened, reperfusion occurred, and the pig was monitored for another 60 min. **B** Coronary anatomy (left) and surgical procedure (right). Exposed heart after a mid-line sternotomy with a silk snare and counter bearing (asterisk) placed around the LAD. RA, right atrium, LA, left atrium, RV, right ventricle, LV, left ventricle, D1, diagonal branch 1, PC, pericardium

After 20 min of stabilization, a midline sternotomy was performed and the LAD was isolated 5 cm away from the apico-septal margin. A ligature was placed around the LAD. The thorax was closed thereafter to reduce artifacts on the ECG recordings and the ligature was tightened through a plastic tube against a counter bearing to induce AMI. Ten minutes after occlusion, 20 ml of either 3 mg/kg amiodarone (amiodaronehydrochloride 50 mg/ml, Paranova, Herlev, Denmark) or saline (Natriumkolorid Fresenius Kabi 9 mg/ml, Fresenius Kabi AB, Uppsala, Sweden) according to the initial randomization was given intravenously into the right ear vein over 5 min. Occlusion was maintained for 120 min followed by a 60-min reperfusion period, performed by opening the ligature (Fig. 1A, B).

### Electrophysiology

A bipolar vector ECG in x-y-z configuration was recorded using three BioAmps (AD Instruments, Dunedin, New Zealand; built-in hardware filter was 0.1–1000 Hz). For the x-lead, electrodes were placed on the left and right sides of the thorax 10 cm dorsal to the sternum and immediately caudal to the front legs. For the y-lead, one electrode was on the manubrium sternum and the other on the *linea alba* 15 cm caudal to the xiphoid. And for the z-lead, electrodes were placed mid-sternal on the dorsal and ventral sides.

Two Franz electrodes (Easy MAP, Föhr Medical Instruments GmbH, Seeheim, Germany) to measure monophasic action potentials (MAP) were placed prior to occlusion via the carotid arterial sheaths in the infarcted apico-septal region and the non-infarcted area on the free wall of the left ventricle (LV) using fluoroscopic guidance (Siremobil Compact L, Siemens, Berlin, Germany). The electrodes were connected to an amplifier (Bio Potential Amp, BPA 79232, Hugo Sacks Elektronik—Harvard Apparatus GmbH, March-Hugstetten, Germany) and signals were amplified 200-fold. All signals were recorded at a 4-kHz sampling rate using AD Instruments PowerLab 16/30 (AD Instruments, Dunedin, New Zealand).

### Hemodynamic Recordings

CO was measured by thermodilution method (REF-1, Baxter International Inc., Deerfield, IL, USA) via the Swan Ganz catheter by injection of 10 ml saline at 4 °C before occlusion, and at every 15 min during occlusion and reperfusion.

### Blood Sampling

Blood sampling was used to monitor acid base homeostasis, electrolytes, blood glucose, and lactate at baseline, before occlusion and every 15–30 min during occlusion and reperfusion. Samples were taken from the arterial access and analyzed with a bedside analyzer (ABL-90, Radiometer, Copenhagen, Denmark). Ventilation was adjusted if necessary.

### Data Analysis

LabChart (Version 8.0, AD Instruments, Dunedin, New Zealand) was used for offline analysis of ECG, MAP and hemodynamic recordings. Primary endpoint of our analysis was freedom from VF and all data was censored after VF occurred.

Four consecutive beats were used to perform manual ECG analysis before occlusion, at minutes 1, 3, 5, 10, and 15 and every 15 min during occlusion and reperfusion. RR, PQ, QRS, and QT intervals and the sum of ST elevation 60 ms after the J point in all three leads were measured during sinus rhythm (SR). ST elevation was calculated as vector sum (ST_*x*_^2^ + ST_*y*_^2^ + ST_*z*_^2^) ^ (1/2). Besides ST elevation, the change in the ST vector magnitude (STC-VM) was calculated as a myocardium at risk estimation according to Näslund et al. [15]. If less than four consecutive ECG complexes in SR were available due to reperfusion arrhythmia or a high number of PVCs, only RR intervals were measured at that time point using ten consecutive beats. MAP recordings from the infarct and non-infarcted areas were analyzed manually and MAP duration at 90% repolarization (MAPD_90_) was measured in SR before occlusion and at minutes 10, 15, and 20 during occlusion. Changes from baseline ΔMAPD_90_ and local beat-to-beat dispersion of MAPD_90_ were calculated. Further, the repolarization rate of MAP was calculated according to MAPD_90_–MAPD_30_ and its beat-to-beat dispersion was calculated. No pacing was applied during acquisition.

ECG was manually revised and PVCs were counted for the first 60 min of coronary occlusion.

### Statistics

All data is reported as mean ± standard error of the mean. Statistical analysis was performed using Prism (Prism 7, GraphPad Software Inc., La Jolla, USA). Comparisons between the two groups in ECG parameters and hemodynamics were evaluated during occlusion using a two-way-ANOVA. Blood gas values, and hemodynamics at baseline and before and after drug injection, STC-VM between the two groups as well as ectopic activity after drug injection were compared for the two groups using Student’s *t* test. The number of animals with alternans after 20 min of occlusion was compared using a chi-square test. VF occurrence was analyzed using a Kaplan-Meier curve and Log-rank testing. *P*-values < 0.05 were considered to be statistically different.

## Results

### Induction of AMI

**Fig. 2:**
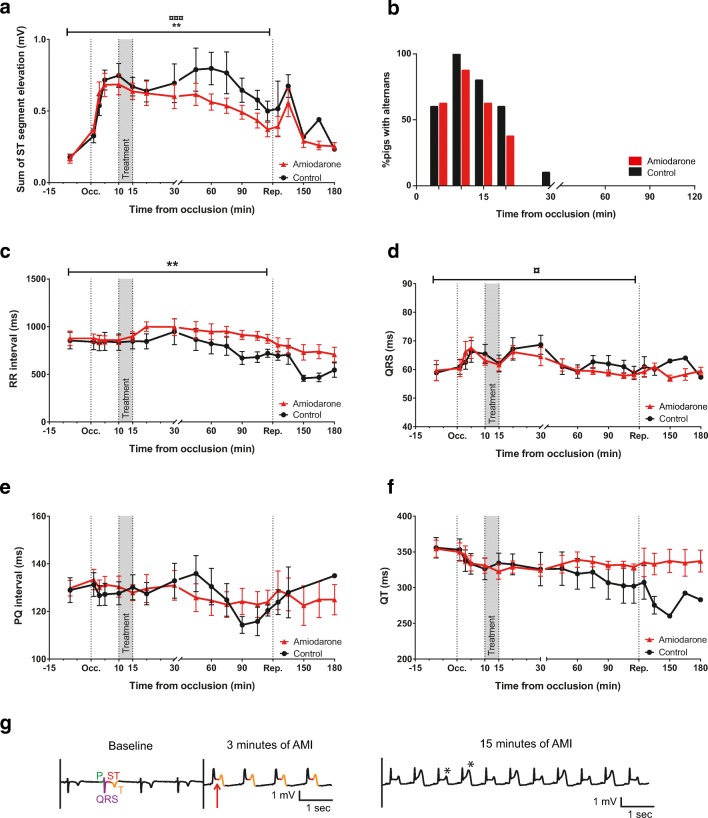
Electrocardiogram (ECG) during acute myocardial infarction (AMI). **A** Mean ST segment elevation plotted over time across 2 h of coronary occlusion of the left anterior descending artery (LAD) and 1 h of reperfusion (^¤¤¤^*P* < 0.0001 for time and **P < 0.005 for treatment, two-way ANOVA). **B** Percentage of pigs with T wave alternans (TWA) was not markedly changed with amiodarone infusion. **C** Longer RR intervals during the occlusion period were observed in the amiodarone group (**P < 0.001 for treatment, two-way ANOVA). **D** QRS duration was affected by AMI (^¤^*p* < 0.05 for time, two-way ANOVA) to the same extent in both groups. **E** and **F** PQ interval and QT interval showed no differences. **G** At baseline, *P* wave (green), QRS complex (purple), ST segment (red), and T wave (orange) are indicated. AMI leads to an elevation of the ST segment (red arrow) and inverted and increased T waves. TWA (asterisk) is present between 5 and 20 min with a maximum after 10–15 min of coronary occlusion. Occ, occlusion, Rep, reperfusion

Induction of AMI with occlusion and subsequent reperfusion was successful in all 18 animals. Occlusion of the LAD caused a rapid elevation in the ST segment within 3 min (Fig. 2A). These effects were comparable in both groups over the first 20 min. A first peak in ST elevation was observed after approximately 10 min (0.69 ± 0.07 mV and 0.74 ± 0.08 mV in the control and the amiodarone group, respectively). Following the initial peak, ST elevation decreased over time during occlusion with a second peak, only present in the control group, at 60 min (0.80 ± 0.11 mV) resulting in an overall difference in ST elevation (*P* < 0.001). A reperfusion peak in ST elevation was observed in both groups.

Beside ST elevation, T wave alternans (TWA) could be observed at some points within the first 20 min of LAD ligation. Amiodarone treatment did not markedly influence TWA (number of pigs with alternans after 20 min of occlusion was 6/10 and 3/8 in the control and amiodarone group, respectively, *P* > 0.05; Fig. 2B). Representative ECG traces at baseline and at 3 and 15 min occlusion are depicted in Fig. 2G.

Occlusion was performed mid-LAD resulting in occlusion sites distally of the second diagonal branch in 4/10 and 3/8 and between the two diagonal branches in 6/10 and 5/8 animals in the control and amiodarone group, respectively. Area at risk was estimated using STC-VM. The mean STC-VM within the first 20 min did not differ in the control and amiodarone group (0.36 ± 0.04 mV and 0.37 ± 0.04 mV, respectively, *P* > 0.05).

### ECG and MAP Changes During AMI

Administration of amiodarone prolonged RR intervals compared to control (*P* < 0.001). AMI resulted in a change of QRS intervals over time (*P* < 0.05) similar in both groups. PQ and QT intervals showed no differences between the groups nor were they affected by AMI (Fig. 2E, F).

**Fig. 3:**
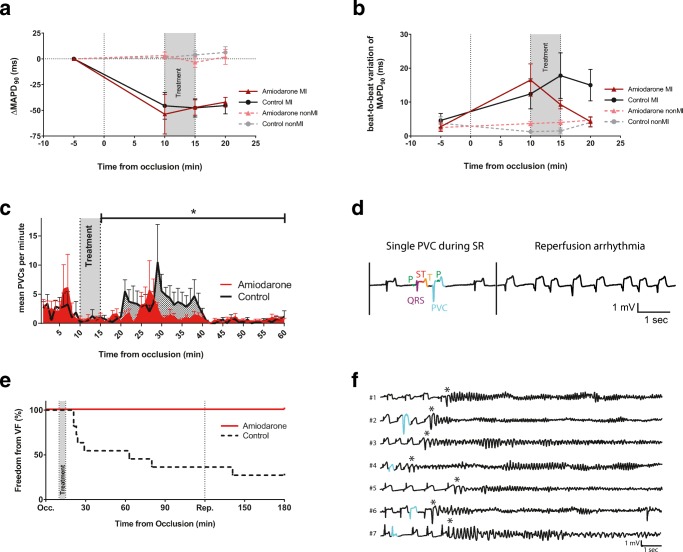
Monophasic action potentials (MAP) and arrhythmias. **A** Duration of MAP at 90% of repolarization compared to baseline (ΔMAPD_90_) during occlusion in the infarcted (MI) and non-infarcted (non-MI) area of the left ventricle. While non-MI MAPD_90_ was stable, MI MAPD_90_ shortened within the first 10 min. **B** Variation of MAPD_90_ due to beat-to-beat alternans increased during myocardial infarction in the MI area. Amiodarone treatment reduced this dispersion. **C** Mean number of premature ventricular contractions (PVC) per minute during the first hour of AMI was lower after amiodarone infusion (*P = 0.013). **D** A single PVC during sinus rhythm (left) and accelerated idioventricular rhythm during reperfusion (right). Retrograde p waves can be seen in all PVCs. **E** Amiodarone was able to prevent ventricular fibrillation (VF) in all pigs when given 10 min after coronary occlusion (*P* < 0.01, Log-Rank test). **F** Sinus rhythm (SR) was present in all pigs before VF occurred. PVC during SR are marked red. Short coupled PVC can be identified as triggering event in all VF episodes (asterisk). Occ, occlusion, Rep, reperfusion

Recordings with MAP electrodes are prone to show a deterioration from MAP-shaped signals to bipolar electrograms due to a loss in electrode/tissue contact over time. A direct measurement of MAPD_90_ from electrogram-shaped signals is not possible. MAP-shaped signals within the first 20 min of AMI could only be recorded for a limited number of electrodes. In the control group, four MAP recordings in the non-infarcted and five in infarcted area, and in the amiodarone group six in the non-infarcted area and four in the infarcted, could be used for analysis. While MAPD_90_ stayed unchanged in the non-infarcted area, it shortened in the infarcted area in both groups. Amiodarone treatment did not notably change MAPD_90_ in any of the two positions (Fig. 3A). AMI led to a dispersion in local repolarization, and to an alternating beat-to-beat variation in action potential shape (repolarization rate MAPD_90_–MAPD_30_) and duration in the infarcted area. Amiodarone reversed this AMI-induced beat-to-beat variation of MAPD_90_ (beat-to-beat variation at 20 min of AMI was 15 ± 5 ms and 4 ± 1 ms in the control and amiodarone group, respectively, Fig. 3B). Dispersion of repolarization rate was 10 ± 2s and 12 ± 3 ms after 10 min of AMI and 17 ± 3 and 3 ± 2 ms after 20 min of AMI for the control and amiodarone groups, respectively.

### Premature Ventricular Contractions and Arrhythmia

The underlying rhythm in all animals was SR with spontaneous PVCs during occlusion. Amiodarone treatment decreased the mean number of PVCs per minute in the 45 min following treatment (1.08 ± 0.17 and 1.97 ± 0.31 in the amiodarone and control group, respectively, *P* = 0.013, Fig. 3C); however, a rather large inter-animal variation was observed.

None of the pigs had ventricular tachycardia during the experiment. One amiodarone-treated animal showed an accelerated idioventricular rhythm with 115 beats per minute starting 24 min after occlusion and returned to SR after 59 min of occlusion. During reperfusion, all animals showed phases with accelerated idioventricular rhythm (Fig. 3D).

### Ventricular Fibrillation During AMI

In the control group, six out of ten pigs developed VF during occlusion and one during reperfusion, compared to the amiodarone group where none of the eight pigs developed VF neither during occlusion nor reperfusion (Fig. 3E). More than half of the VF episodes occurred between 20 and 30 min and all of them were triggered by PVCs (Fig. 3F). Further, all VF episodes were sustained and required defibrillation. Before onset of VF, all animals were in SR. None of the animals that developed VF developed cardiogenic shock prior to onset of VF (Table 1).

**Table 1:** Characteristics of animals with ventricular fibrillation

Animal	Time to VF (min)	Heart rate before VF (min^−1^)	Arterial blood pressure systolic/diastolic (mean), (mm Hg)	Lactate (mmol/L)
#1	80	63	87/48 (61)	1.3
#2	29	80	71/55 (60)	1.0
#3	24	94	61/35 (44)	0.7
#4	21	97	80/47 (58)	1.1
#5	63	54	79/45 (56)	2.3
#6	143	65	78/42 (54)	1.9
#7	21	80	74/47 (56)	1.3

VF: ventricular fibrillation, mm Hg: millimeter of mercury

### Hemodynamics and Blood Gas

**Fig. 4:**
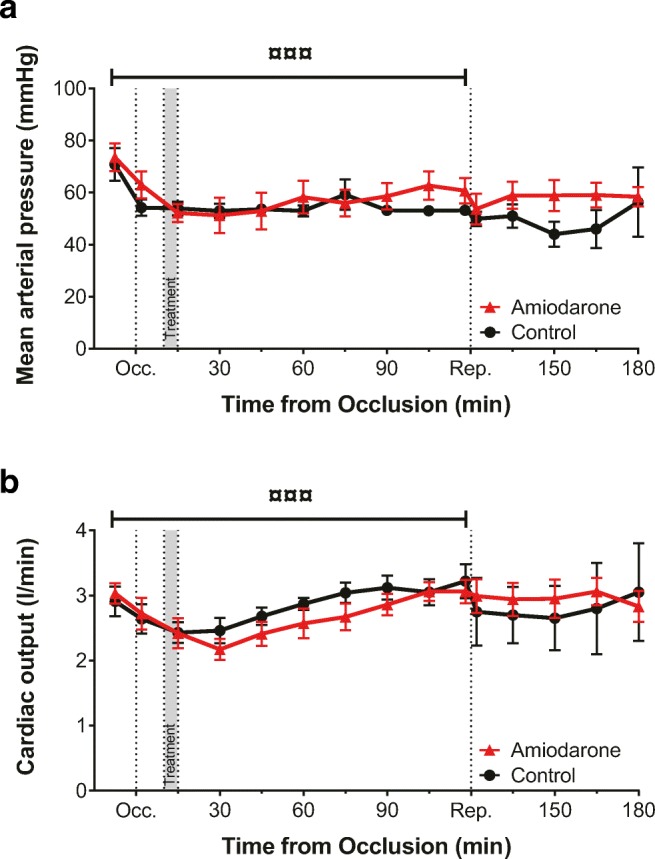
Hemodynamic parameters*.* Mean arterial blood pressure (**A**) and cardiac output (**B**) were analyzed as safety parameters. Coronary occlusion caused a drop in all hemodynamic parameters, but none of the hemodynamic parameters was affected by amiodarone treatment (^¤¤¤^*P* < 0.0001 for time, two-way ANOVA). Occ, occlusion, Rep, reperfusion

AMI led to a comparable drop in mean arterial pressure and CO in all animals over time for both groups during occlusion (*P* < 0.0001 for time and *P* > 0.05 for group differences; Fig. 4A, B). Thus, amiodarone treatment did not affect hemodynamic stability compared to surviving controls.

Stable hemodynamics was also reflected in blood gas parameters. These were comparable in the amiodarone and saline groups at baseline, before and 5 min after drug injection (data not shown).

## Discussion

Amiodarone infused 10 min after onset of AMI induced via mid-LAD occlusion prevented occurrence of VF, which happened at a high proportion in the AMI control group.

Positive effects of amiodarone administered prior to AMI on the occurrence of VF have previously been shown. In a dog model of chronic anterior wall infarction, acute occlusion of the left circumflex artery (LCx) was performed after a treatment with amiodarone with 10 mg/kg/h intravenously for 2 h, starting 1 h before LCx infarction (short-term) or 10 mg/kg/day orally over 24 days (long-term) before LCx infarction. A reduction in VF incidence from 100% in the control to 60% in the amiodarone group for short-term treatment and an even stronger effect for the long-term treated dogs was reported [16]. Prophylactic treatment with amiodarone (3 mg/kg) and lidocaine given 20 min prior to AMI in sheep resulted in reduced incidence of arrhythmia and mortality rate [17].

Our results are in line with those findings, even though in our experiments, amiodarone was given 10 min after coronary occlusion, approximately 15 min before the first VF cases occurred. Although the design of our study resembles other studies on amiodarone and AMI, our study is the first to show amiodarone to be effective when given after coronary occlusion. This is an important fact as the temporal relationship between drug admission and coronary occlusion not only plays a role for translatability into the clinics but also can abolish antiarrhythmic effects of a drug or even provoke proarrhythmic effects as shown by Nattel et al. for aprindine [18]. As amiodarone is infused after occlusion, we do not expect the compound to be present at a high concentration in the ischemic area. However, in spite of this, the compound can protect against arrhythmias, indicating that stabilization of the border zone and normal perfused myocardium is enough to depress occurrence of arrhythmia.

### ECG and MAP

AMI-induced elevation of the ST segment was observed within the first minute and showed a similar kinetic and magnitude in both groups during the first 30 min of AMI. A second peak in ST elevation could be observed in the control group 60 min after coronary occlusion that was not present in the amiodarone group. Literature on ST elevation kinetics during AMI is scarce and does not provide pathophysiological theories on underlying mechanisms [15]. Amiodarone treatment in patients with angina pectoris has been shown to reduce ST depression during exercise testing [19]. Since amiodarone previously has been found to work both as a direct vasodilator [20] and by adrenergic antagonism [21], the absence of the second ST elevation peak in the amiodarone group could be related to one of these effects. As diastolic blood pressure is proportional to coronary perfusion pressure, we assumed that a lower diastolic pressure would result in less perfusion of the border zone of the infarcted area thereby increasing local ischemia seen as ST elevation in the ECG. Our data showed a weak negative correlation between ST elevation and diastolic pressure in the control group (*R*^2^ = 0.36, *P* = 0.004) but not in the amiodarone group (*R*^2^ = 0.02, *P* = 0.423) during the second peak. Amiodarone-induced coronary vasodilation seems therefore likely to ensure better perfusion of the infarct border zone at lower perfusion pressures.

We observed a reduction of heart rate after amiodarone was infused. No effect with amiodarone was found on PQ intervals, QRS duration, and QT interval during coronary occlusion. Changes in QT intervals during acute treatment can occur, but are claimed to be rare [10]. AMI itself resulted in a shortening of QT duration within the first 10 min.

In the ischemic area, MAPD_90_ shortened within the first minutes of AMI in the control and amiodarone groups, while MAPD_90_ remained unchanged in the non-infarcted area. These local differences in APD lead to a dispersion in repolarization. Several experiments on the effect of acute amiodarone administration on APD in a variety of animal models and isolated cardiomyocytes have been performed with conflicting findings; however, most of these showed a moderate prolongation of APD [10]. A study conducted in 24 dogs investigated the acute effect of amiodarone on MAPD_90_ in a dose dependent manner after coronary occlusion of the LAD. Mayuga and Singer found that amiodarone treatment with 10 mg/kg was able to counteract MAPD_90_ shortening in the infarcted area and thereby decrease dispersion in repolarization. This resulted into a lowered vulnerability to programmed ventricle stimulation in the infarcted and non-infarcted areas [22]. In our experiments, we did not observe a difference in MAPD_90_ between the two groups. This may be explained by the low dose of amiodarone (3 mg/kg) or the varying RR intervals during SR.

Alternans in local electrograms is a known indicator of electrical instability and poses a risk factor for VF [23]. In our study, amiodarone was able to reduce the beat-to-beat variation of MAPD_90_ and MAP morphology (repolarization rate MAPD_90_—MAPD_90_) in the infarcted area. Overall TWA in the surface ECG was not notably affected by treatment.

Another suspected mechanism in arrhythmia prevention is increased ERP. Whether an increase in ERP mediated by amiodarone is already present at this early phase of AMI when arrhythmias occur is debatable. Tsagalou et al. measured ERP in pigs treated with amiodarone (5 mg/kg) after coronary occlusion. Due to AMI ERP shortened in the untreated control group while animals treated with amiodarone experienced a prolongation of their ERP after 30 min [11]. Since our aim was to study the effect of amiodarone on VF occurrence, we abstained from pacing and measuring ERP during our own experiments to avoid interfering with the natural cause of the arrhythmia.

### Premature Ventricular Contractions and Arrhythmias

PVCs occurred in two distinct phases: The first within the first minutes of coronary occlusion, the second after approximately 20 min of coronary occlusion. TWA usually occurred in phases of low ectopic activity. Analysis of arrhythmias showed a reduction in PVCs over 45 min following treatment. These findings are in accordance with others who describe effects on the occurrence of PVCs 15 to 30 min after amiodarone treatment [13, 16]. A reduction of triggering factors as a protective mechanism against VF in this early phase of AMI seems therefore likely.

### Effect on Hemodynamics

The induced AMI resulted in only moderate hemodynamic alterations without leading to cardiogenic shock. In our experiments, amiodarone did not negatively influence hemodynamic parameters compared to the control group. Mean arterial blood pressure was not affected by the treatment, neither was CO. This is in accordance with findings from studies on hemodynamic effects of amiodarone in dogs [24].

### Limitations

The focus on spontaneous occurrence of VF led to some limitations. We omitted programmed electrical stimulation for measuring ERP during the procedure to avoid accidentally VF induction during AMI. MAP electrodes were placed right after the coronary ligature was placed, but before it was tightened. Nevertheless, the signal obtained deteriorated fast leaving only a few animals left for actual analysis of MAPD_90_. All MAPD_90_ were measured during SR without further correction for heart rate. MAP electrodes have a low spatial resolution and MAPD90 or beat-to-beat variation of MAPD90 represents the electrophysiological properties at the electrode tip. Whole heart mapping techniques could have provided a more integrated picture of the electrophysiological properties and origins of PVCs.

We only used indirect methods (occlusion site, STC-VM) to determine the myocardium at risk during the coronary occlusion and did not measure infarct size at the end of the experiments. Amiodarone could potentially have had an impact on infarct size as it has vasodilating effects and beta-blocking properties. However, Li et al. investigating infarct size and acute amiodarone treatment in sheep reported similar infarct sizes for control and intervention groups [17]. In dogs, amiodarone treatment led to a decrease in infarct size, presumably by an increase in collateral blood flow in the infarcted area [25]; however, as pigs are lacking coronary collateral flow, this mechanism might be of minor importance.

### Clinical Perspectives

The results from this study can potentially be utilized to perform a randomized clinical trial. We selected the time from coronary occlusion to treatment to be 10 min, representing the time for emergency medical services to arrive, obtain an ECG, and diagnose STEMI; however, a possible clinical trial investigating the effect of amiodarone in an unselected STEMI cohort has to include high number of patients. Based on data from the e-MUST [5] trial and our GEVAMI study [4], we estimate a sample size of 3084 (1542 patients per group) to provide 90% power to detect an absolute risk reduction of 50% in the incidence of VF during transport or procedure. This results in a high number of patients needed to treat and emphasizes the need for a better risk stratification to identify patients at risk for VF.

### Conclusion

We were able to show that amiodarone given after onset of AMI could prevent the occurrence of VF and subsequent sudden cardiac death in a porcine model. We identified a reduction in heart rate, a reduced number of PVCs per minute and a reduced local dispersion of MAPD_90_ with amiodarone as putative protective factors. Hemodynamic parameters were not influenced by amiodarone treatment.

Before these findings can be translated into a clinical trial, additional risk assessment tools in the early phase of AMI are necessary to identify patients at risk of VF which potentially will gain from early amiodarone treatment.
